# Supplementary figures and images for: SSREnricher: a computational approach for large-scale identification of polymorphic microsatellites based on comparative transcriptome analysis
Source: PeerJ. 2020 Jul 2;8:e9372. doi: 10.7717/peerj.9372 (PMC7335497; doi:10.7717/peerj.9372)

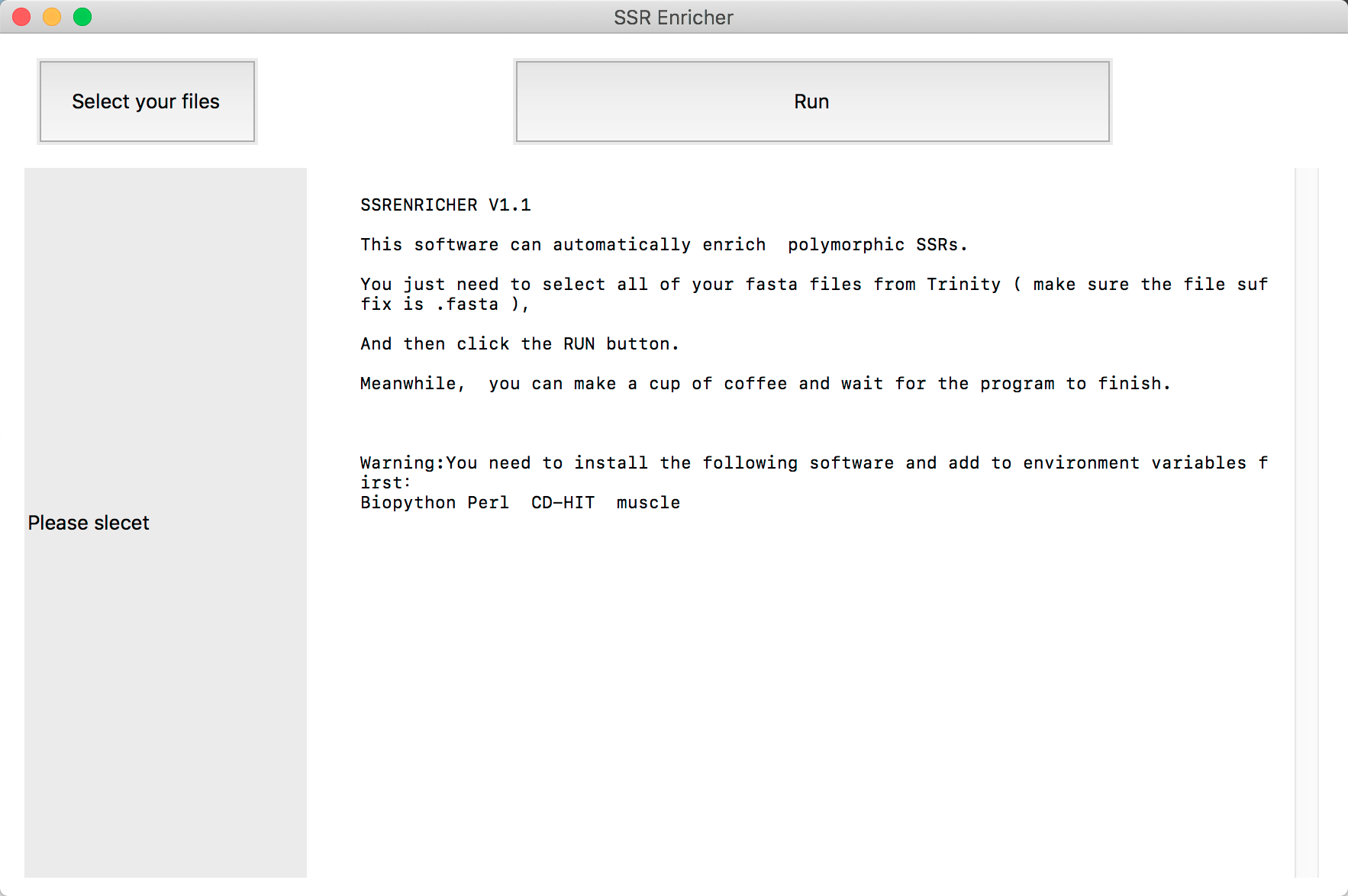

Supplement: Supplemental Information 4 [file peerj-08-9372-s004.zip › GUI.png]

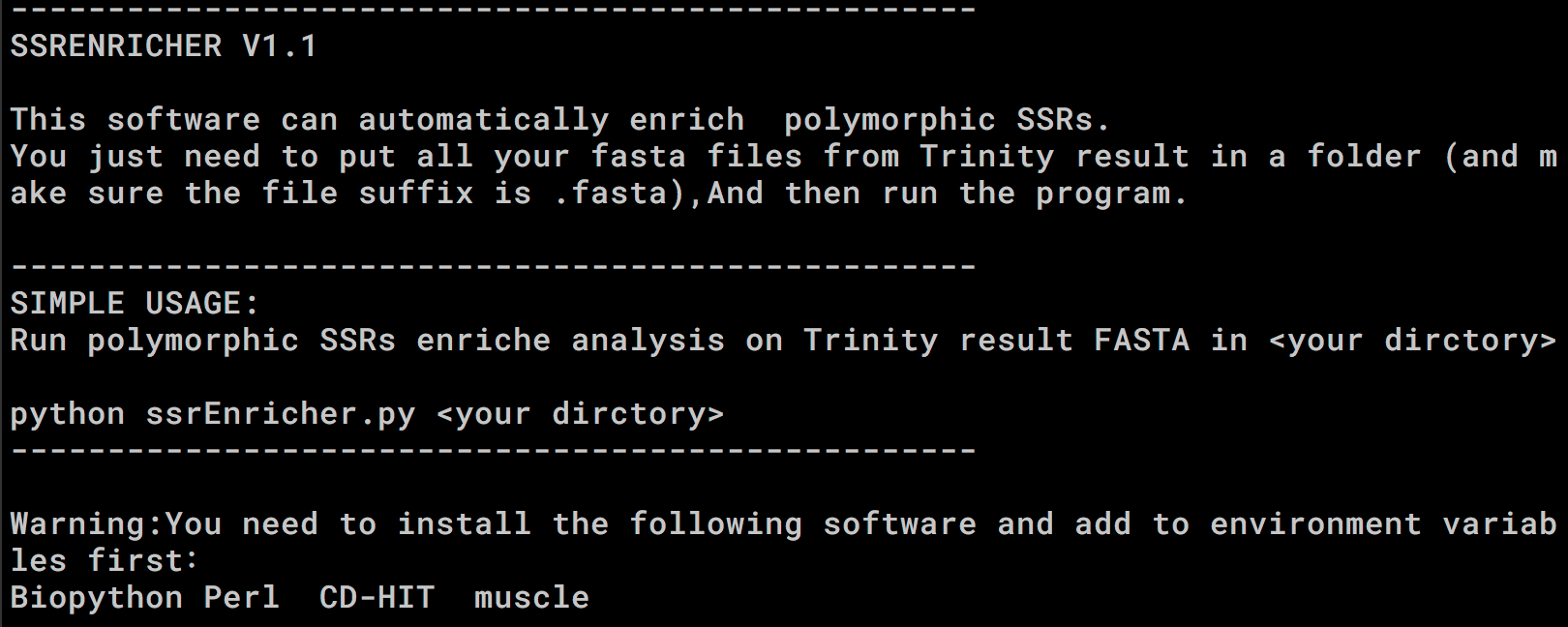

Supplement: Supplemental Information 4 [file peerj-08-9372-s004.zip › comandLine.png]

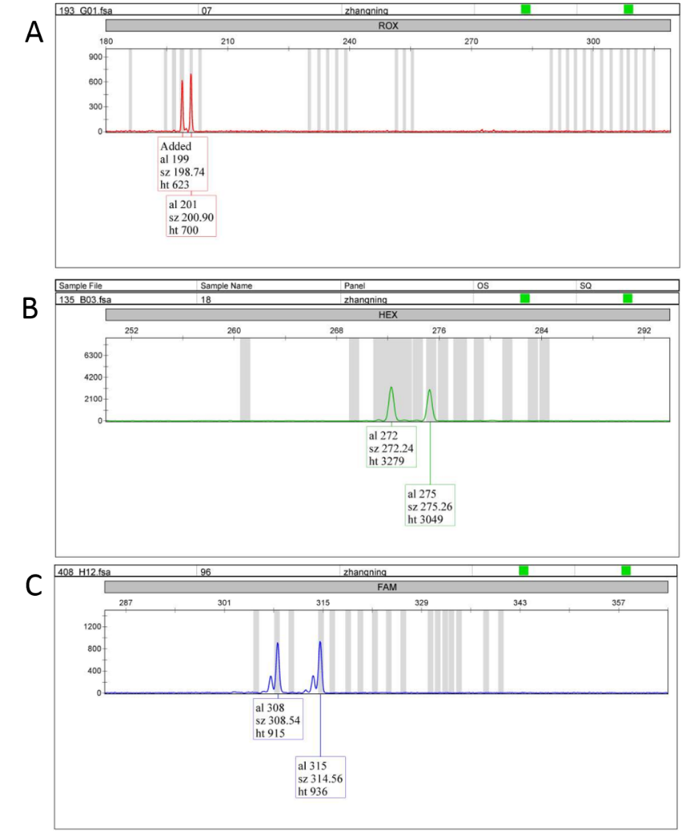

Supplement: Supplemental Information 5 — A, SSR Ci-SSR28, the primer of which was labeled with ROX; B, SSR Ci-SSR26, the primer of which was labeled with HEX; C, SSR Os-SSR29, the primer of which was labeled with FAM. [file peerj-08-9372-s005.zip › electrophoresis.png]
